# Supplementary material for: Analysis of PPARγ Signaling Activity in Psoriasis
Source: Int J Mol Sci. 2021 Aug 10;22(16):8603. doi: 10.3390/ijms22168603 (PMC8395241; doi:10.3390/ijms22168603)
Supplement: Supplementary file 1 [file ijms-22-08603-s001.zip › Supplemental materials_Analysis of PPARg signaling activity in psoriasis/Pathway models/Models images and html files/Differentiation of psoriatic T cells/6189.html]

IFNAR1


# Protein IFNAR1

|  |  |
| --- | --- |
| URN | urn:agi-llid:3454 |
| Total Entities | 0 |
| Connectivity | 1196 |
| Name | IFNAR1 |
| Description | interferon alpha and beta receptor subunit 1 |
| Notes | The protein encoded by this gene is a type I membrane protein that forms one of the two chains of a receptor for interferons alpha and beta. Binding and activation of the receptor stimulates Janus protein kinases, which in turn phosphorylate several proteins, including STAT1 and STAT2. The encoded protein also functions as an antiviral factor. [provided by RefSeq, Jul 2008] |
| Primary Cell Localization | Plasma membrane |
| Class | Receptor |
| ObjectType | Protein |

---

|  |  |
| --- | --- |
| Pathway | AXL Receptor Inhibits Macrophages and Dendritic Cells Function |
|  | Proteins Involved in Myocarditis |
|  | SOCS in Negative Feedback Regulation in Myocarditis |
|  | T-Cells Differentiation Block in Psoriasis |
|  | Dendritic Cell Activation in Systemic Lupus Erythematosis |
|  | Proteins Involved in Systemic Lupus Erythematosus |
|  | Th17-Cell Function in Systemic Lupus Erythematosus |
|  | B-Cell Activation in Systemic Lupus Erythematosus |
|  | Dendritic Cell Dysfunction in Psoriatic Arthritis |
|  | N2 Neutrophils in Tumor-Promoting Inflammation and Tumor Progression |
|  | IFNW1/IFNR Expression Target |
|  | IFNA1/IFNR Expression Targets |
|  | IFNB1/IFNR Expression Targets |
|  | AXL Receptor Tyrosine Kinase Signaling |
|  | PPAR Psoriasis |
|  | New Pathway (3) |
|  | dsRNA induces innate immunity and INFs in alveolar cells |
|  | IFN signaling in alveolar cells |
|  | Dendritic Cell Activation in COVID19 |
|  | 1\_Differentiation of psoriatic T cells |
|  | drug-target pairs binding |
|  | drug-target pairs direct regulation |
|  | Differentiation of psoriatic T cells |

---

|  |  |
| --- | --- |
| MedScan ID | 3454 |

---

|  |  |
| --- | --- |
| LocusLink ID | 3454 |
|  | 15975 |
|  | 288264 |

---

|  |  |
| --- | --- |
| Alias | INF-alpha receptor |
|  | interferon receptor 1 |
|  | interferon (alpha, beta and omega) receptor |
|  | interferon (alpha and beta) receptor I |
|  | OTTMUSP00000033798 |
|  | INF-a receptor |
|  | IFRC |
|  | cytokine receptor class-II member 1 |
|  | Infar |
|  | interferon alpha/beta receptor 1 |
|  | human interferon-alpha receptors |
|  | Hu-IFN-alphaR1 |
|  | interferon-alpha receptor I |
|  | OTTMUSP00000033797 |
|  | LOC284829 |
|  | INF-alpha receptors |
|  | INF-a receptors |
|  | IFNAR |
|  | CRF2-1 |
|  | IFN-alpha-R1 |
|  | alpha-type antiviral protein |
|  | Ifar |
|  | type I interferon receptor 1 |
|  | interferon (alpha, beta and omega) receptor 1 |
|  | cytokine receptor family 2 member 1 |
|  | IFNBR |
|  | interferon-beta receptor 1 |
|  | Ifnar1 |
|  | IFN-R-1 |
|  | beta-type antiviral protein |
|  | human interferon-alpha receptor |
|  | INR1 |
|  | interferon alpha and beta receptor subunit 1 |
|  | IFN alpha R |
|  | human interferon-alpha receptor (HuIFN-alpha-Rec) |
|  | INR1\_HUMAN |
|  | interferon (alpha, beta and omega) receptor I |
|  | HuIFN-alphaR1 |
|  | interferon-alpha receptor 1 |
|  | Ifnar I |
|  | IFN-alpha-REC |
|  | CD118 |
|  | interferon (alpha and beta) receptor 1 |
|  | HuIFN-alpha-Rec |
|  | Interferon-alpha/beta receptor alpha chain |
|  | IFN-R |
|  | interferon-beta receptor I |
|  | IFN-alpha/beta receptor 1 |
|  | IFN-alpha/betaR |
|  | INR I |
|  | AVP |
|  | interferon-alpha/beta receptor 1 |

---

|  |  |
| --- | --- |
| GO ID | 0004896 |
|  | 0019962 |
|  | 0004905 |
|  | 0007259 |
|  | 0035457 |
|  | 0019221 |
|  | 0032496 |
|  | 0009615 |
|  | 0060337 |
|  | 0005887 |
|  | 0005770 |
|  | 0005764 |
|  | 0005886 |
|  | 0004904 |
|  | 0004637 |
|  | 0042110 |
|  | 0051607 |
|  | 0032728 |
|  | 0032729 |
|  | 0050718 |
|  | 0045893 |
|  | 0050730 |
|  | 0045351 |
|  | 0005768 |
|  | 0016021 |
|  | 0016020 |
|  | 0060759 |
|  | 0007166 |
|  | 0060338 |

---

|  |  |
| --- | --- |
| KEGG ID | hsa:3454 |
|  | mmu:15975 |
|  | rno:288264 |

---

|  |  |
| --- | --- |
| Organism | Homo sapiens {Organism urn:agi-taxid:9606} |
|  | Mus musculus {Organism urn:agi-taxid:10090} |
|  | Rattus norvegicus {Organism urn:agi-taxid:10116} |
|  | Mus musculus |
|  | Homo sapiens |
|  | Rattus norvegicus |

---

|  |  |
| --- | --- |
| Mouse chromosome position | 16 52.98 cM |
|  | 16 63.2 cM |

---

|  |  |
| --- | --- |
| OMIM ID | 107450 |

---

|  |  |
| --- | --- |
| Rat chromosome position | 11q11 |

---

|  |  |
| --- | --- |
| Hugo ID | 5432 |
|  | HGNC:5432 |

---

|  |  |
| --- | --- |
| Human chromosome position | 21q22.11 |

---

|  |  |
| --- | --- |
| Swiss-Prot Accession | P17181 |
|  | P17181.3 |
|  | P33896 |
|  | P33896.2 |
|  | D3ZDS9 |
|  | A0A0G2JXD8 |
|  | B2R6L9 |
|  | B4DNT3 |
|  | D3DSF0 |
|  | Q53GW9 |
|  | Q53H11 |
|  | Q6PKD7 |
|  | Q7M4L2 |
|  | Q8WTZ2 |
|  | Q80UJ3 |

---

|  |  |
| --- | --- |
| PIR ID | A32694 |
|  | S41602 |
|  | A45283 |

---

|  |  |
| --- | --- |
| GenBank ID | NC\_000021 |
|  | XM\_005260964 |
|  | XP\_005261021 |
|  | XM\_011529552 |
|  | XP\_011527854 |
|  | NM\_000629 |
|  | NP\_000620 |
|  | AF039907 |
|  | AP000296 |
|  | AP000297 |
|  | AP000298 |
|  | AP001716 |
|  | AY654286 |
|  | AAT49100 |
|  | CH471079 |
|  | EAX09837 |
|  | EAX09838 |
|  | EAX09839 |
|  | KF510545 |
|  | X60459 |
|  | CAA42992 |
|  | AK123813 |
|  | AK222770 |
|  | BAD96490 |
|  | AK222812 |
|  | BAD96532 |
|  | AK298051 |
|  | BAG60345 |
|  | AK312631 |
|  | BAG35516 |
|  | BC002590 |
|  | AAH02590 |
|  | BC014338 |
|  | BC021825 |
|  | AAH21825 |
|  | BC035603 |
|  | BC065751 |
|  | BG116121 |
|  | BG548299 |
|  | BX537636 |
|  | CA309858 |
|  | J03171 |
|  | AAA52730 |
|  | JN543953 |
|  | AFN73226 |
|  | JN543954 |
|  | AFN73227 |
|  | JN543955 |
|  | AFN73228 |
|  | JN543956 |
|  | AFN73229 |
|  | R15745 |
|  | P17181 |
|  | NC\_000082 |
|  | XR\_384931 |
|  | NM\_010508 |
|  | NP\_034638 |
|  | AC132272 |
|  | AC150035 |
|  | CH466602 |
|  | EDL03825 |
|  | EDL03826 |
|  | EDL03827 |
|  | U06237 |
|  | AAA65003 |
|  | U06238 |
|  | AAC01749 |
|  | U06239 |
|  | AAA65004 |
|  | U06240 |
|  | AAA65005 |
|  | U06241 |
|  | AAA65006 |
|  | U06242 |
|  | AAA65007 |
|  | U06244 |
|  | AAA65008 |
|  | AK040184 |
|  | AK132431 |
|  | BAE21165 |
|  | AK136950 |
|  | AK141630 |
|  | BAE24775 |
|  | AK148246 |
|  | BAE28435 |
|  | AK153583 |
|  | BAE32110 |
|  | AK157555 |
|  | AK178895 |
|  | AK193062 |
|  | AK217677 |
|  | BC043935 |
|  | AAH43935 |
|  | BC052217 |
|  | AAH52217 |
|  | BC052429 |
|  | AAH52429 |
|  | BE955685 |
|  | CA322032 |
|  | M89641 |
|  | AAA37890 |
|  | P33896 |
|  | NC\_005110 |
|  | NM\_001271316 |
|  | NP\_001258245 |
|  | NM\_001105893 |
|  | NP\_001099363 |
|  | XM\_017597943 |
|  | XP\_017453432 |
|  | XM\_017597942 |
|  | XP\_017453431 |
|  | AC\_000079 |
|  | AAHX01068280 |
|  | AAHX01068281 |
|  | AC120974 |
|  | CH473989 |
|  | EDM10721 |
|  | EDM10722 |
|  | EDM10723 |
|  | EDM10724 |
|  | FQ210936 |
|  | FQ218374 |
|  | FQ235261 |
|  | AAHY01130191 |
|  | ABM82249 |
|  | AMYH02037735 |
|  | AC\_000038 |
|  | DQ891323 |
|  | AABR06067655 |
|  | AC\_000153 |
|  | NC\_018932 |
|  | ABBA01057338 |
|  | ABM85433 |
|  | DQ894507 |

---

|  |  |
| --- | --- |
| Swiss-Prot ID | INAR1\_HUMAN |
|  | INAR1\_MOUSE |

---

|  |  |
| --- | --- |
| Cell Localization | Cell membrane |
|  | Late endosome |
|  | Lysosome |
|  | Membrane |
|  | Plasma membrane |

---

|  |  |
| --- | --- |
| Ensembl ID | ENSG00000142166 |
|  | ENSP00000270139.3 |
|  | ENST00000270139.8 |
|  | ENSP00000498654.1 |
|  | ENST00000652450.1 |
|  | ENSP00000498491.1 |
|  | ENST00000652513.1 |
|  | ENSMUSG00000022967 |
|  | ENSMUSP00000023689.4 |
|  | ENSMUST00000023689.10 |
|  | ENSMUST00000123196.8 |
|  | ENSRNOG00000028594 |
|  | ENSRNOP00000031611.5 |
|  | ENSRNOT00000029985.6 |
|  | ENSRNOP00000070232.1 |
|  | ENSRNOT00000091472.1 |
|  | ENST00000270139.7 |
|  | ENSRNOP00000070232 |
|  | ENSMUST00000023689 |
|  | ENSMUSP00000023689 |
|  | ENSP00000270139 |
|  | ENSRNOT00000029985 |
|  | ENST00000270139 |
|  | ENSRNOT00000091472 |
|  | ENSRNOP00000031611 |

---

|  |  |
| --- | --- |
| MGI ID | MGI:107658 |
|  | 107658 |

---

|  |  |
| --- | --- |
| RGD ID | 1305399 |

---

|  |  |
| --- | --- |
| TCDB ID | 8.A.47.2.1 |

---

|  |  |
| --- | --- |
| Unigene ID | Mm.502 |
|  | Hs.529400 |
|  | Rn.105738 |

---

|  |  |
| --- | --- |
| Homologene ID | 524 |

---

|  |  |
| --- | --- |
| Shape | Stick-vertex |

---

|  |  |
| --- | --- |
| IPI ID | IPI00115420 |
|  | IPI00877162 |
|  | IPI00877141 |
|  | IPI00012877 |

---
